# Local and systemic immune correlates of anal high-risk HPV infection and clearance in men living with HIV and men at high risk for HIV

**DOI:** 10.3389/fmicb.2026.1855627

**Published:** 2026-06-25

**Authors:** Veronica Ober, Eva Gruener, Danni Wang, Olga Baranov, Renate Stirner, Gabriele Reiling, Raffaele Conca, Christopher Dächert, Gerardo Ibarra, Ulrich Seybold, Johannes R Bogner, Christof Geldmacher, Kathrin Held, Julia Roider

**Affiliations:** 1 Department of Infectious Diseases, Department of Medicine IV, LMU University Hospital, LMU Munich, Munich, Germany; 2 German Centre for Infection Research, Partner Site Munich, Munich, Germany; 3 Institute of Infectious Diseases and Tropical Medicine, LMU University Hospital, LMU Munich, Munich, Germany; 4 Department of Pediatrics, Dr. Von Hauner Children’s Hospital, LMU University Hospital, LMU Munich, Munich, Germany; 5 Max Von Pettenkofer Institute, Virology, National Reference Center for Retroviruses, Faculty of Medicine, LMU Munich, Munich, Germany; 6 Fraunhofer Institute for Translational Medicine and Pharmacology ITMP, Immunology, Infection and Pandemic Research, Munich, Germany; 7 Unit Global Health, Helmholtz Zentrum München, German Research Centre for Environmental Health (HMGU), Neuherberg, Germany

**Keywords:** HPV infection, immune correlates, men living with HIV, men’s health, T cell response

## Abstract

**Introduction:**

Human papillomavirus (HPV) prevalence is high among men who have sex with men (MSM), increasing the risk of anal cancer. Key risk factors include HIV co-infection and persistent HPV infection, although the underlying mechanisms remain unclear.

**Methods:**

We characterized HPV burden in 75 men (median age 53 years [IQR 41–60]) presenting to LMU University Hospital or the medical practice prinzmed between 2022 and 2024. 64/75 defined themselves as MSM, 49 MSM were living HIV and 15 without HIV. 11/75 non-MSM were living with HIV. To identify systemic immunological correlates of HPV clearance or persistence, we performed IFN-γ ELISpot and peripheral blood T cells were analyzed by flow cytometry. Further, in a subgroup ano-mucosal CD8^+^ T cells were profiled via low-input RNA sequencing to determine immune signatures associated with persistent HPV infection.

**Results:**

Anal HPV infection was highly prevalent at baseline (67/75) in this largely unvaccinated cohort (73/75), particularly among MSM (59/64), and was independent of HIV status. At baseline, simultaneous infection with multiple HPV types was detected in 50/64 MSM versus 4/11 non-MSM, and most infections persisted over 1 year. Type-specific systemic T-cell responses were detected in 3/4 individuals who cleared a given HR HPV type, compared with 0/14 with persistent HR HPV (*p* = 0.049). Among men living with HIV, HR HPV clearance was lower compared to men without HIV. Peripheral T cells in men living with HIV and infected with at least one HR HPV strain exhibited markers of exhaustion (TIM-3^+^ CD4^+^ and CD8^+^ T cells [*p*-value = 0.01 for CD4^+^ and *p*-value = 0.04 for CD8^+^]; Tcf1^–^PD-1^+^TIM-3^+^ CD4^+^ T cells [*p*-value = 0.02]), and senescence (CD57^+^ CD4^+^ T cells, *p*-value = 0.02). Ano-mucosal CD8^+^ T cells of HR HPV infected men living with HIV showed upregulation of genes associated with exhaustion (*CTLA4, CD101*), activation and effector function (*GZMK, CTSW*) compared to non-HR HPV infected men.

**Discussion:**

Our findings show a high anal HPV burden and low clearance among MSM, regardless of HIV status. Clearance was associated with type-specific systemic T-cell responses, while T-cell exhaustion and senescence may contribute to reduced clearance in men living with HIV.

## Introduction

Human papillomavirus (HPV) infection is highly prevalent among both men and women, and represents a substantial global health burden due to its association with a variety of epithelial malignancies at the site of infection. While HPV is widely recognized as the causative agent of cervical cancer in women, it also plays a crucial role in men’s health as implicated in several other malignancies, including oropharyngeal cancer, head and neck squamous cell carcinoma (HNSCC), and anal cancer (Scott-Wittenborn and Fakhry, 2021; Moscicki et al., 2012).

Human papillomavirus genotypes are classified according to their oncogenic potential into low-risk (LR) and high-risk (HR) types. LR HPV types are primarily associated with benign lesions such as genital warts. In contrast, HR HPV genotypes, e.g., HPV16, 18, 35, 45 and 58, are capable of inducing intraepithelial lesions that may progress to invasive carcinoma (Scott-Wittenborn and Fakhry, 2021). For certain HPV types [probably or possibly cancerogenic high-risk (PHR)], the carcinogenic potential remains incompletely defined or has only been observed under certain conditions like immunosuppression (de Martel et al., 2020).

Human papillomavirus is transmitted predominantly through sexual intercourse and skin-to-skin contact. Anal HPV infection has a particularly high burden in men who have sex with men (MSM), in whom prevalence reaches 50%–100% (Zhou et al., 2021; Supindham et al., 2015; Marra et al., 2019; van Aar et al., 2013; Nyitray et al., 2011). Factors contributing to the elevated prevalence in MSM include a higher average number of lifetime sexual partners (Daling et al., 2004), preferred sexual practice (Chin-Hong et al., 2004; Wei et al., 2023) and low vaccination coverage, as men have historically been underrepresented in HPV vaccination strategies (Loke et al., 2017).

While most HPV infections are cleared spontaneously within 12 months (Moscicki et al., 2004; van Aar et al., 2014), persistence of HR HPV infection is a key determinant of malignant progression and significantly increases the risk of HPV-associated cancers (Brickman and Palefsky, 2015).

Men who have sex with men living with HIV and HPV exhibit significantly lower HPV clearance compared to MSM without concomitant HIV infection, resulting in an increased risk of cancer development at the site of HPV infection (Looker et al., 2018; Mooij et al., 2014; van Aar et al., 2014). Several pathogenic mechanisms have been proposed to account for the impaired clearance of HPV in people living with HIV. In addition to chromosomal instability observed in HIV/HPV co-infection and transcriptional interactions between the two viruses (Zhang et al., 2025; Palefsky, 2006; Tornesello et al., 1993), immunological alterations - both local at the site of infection and systemic - are thought to critically influence the course of HPV infection and, consequently, increase cancer risk (Tong et al., 2015; Brickman and Palefsky, 2015; Guo et al., 2023; Palefsky, 2006).

Local immune dysregulation, including HIV-driven depletion of CD4^+^ T cells in the gastrointestinal tract and compromised epithelial integrity resulting from chronic pro-inflammatory signaling (Strickler et al., 2005; Tugizov, 2016; Piketty et al., 2013), persists despite effective antiretroviral treatment (ART). Moreover, systemic antiviral immune responses to HPV may be further impaired by dysfunctional CD8^+^ T cell responses in individuals living with HIV (Krishna et al., 2018; Mbuya et al., 2021).

In this study we characterized the burden of HPV infection in a German cohort of 75 men in relation to sexual behavior patterns and HIV co-infection. We further assessed the relationship of systemic HPV-specific immune responses with HPV clearance, and characterized alterations in anal and systemic immune cell populations during HIV/HPV co-infection to advance the understanding of HPV-related risk factors and the immunological dynamics in HIV/HPV co-infection.

## Materials and methods

### Study design and approval

This observational, longitudinal follow-up study was approved by the Institutional Review Board of Medical Faculty at Ludwig-Maximilians-University Munich (LMU; project number 19-0849). All participants in this study were men, of whom 64/75 identified as MSM (Table 1). Men living with HIV and men living without HIV obtaining routine medical care at the outpatient clinic at LMU Hospital or at the medical practice prinzmed were invited to participate in our study. After obtaining written informed consent, sampling included two anal swabs and EDTA-blood. Men living with HIV were sampled thrice (week 13 [IQR 13–15], 27 [IQR 25–28] and 53 [IQR 51–55]), while men living without HIV were sampled twice (week 35 [IQR 31–45] and 53 [52–56]) after baseline visits from 2022 to 2024 (Table 2). The differences in sampling time points between men living with and without HIV reflect the differing schedules of routine clinical visits in the two groups. Detailed clinical characteristics of individuals included in this study and living with HIV are summarized in Supplementary Table 1.

**TABLE 1:** Study characteristics.

	PLWH (*n* = 60)	PLWoH (*n* = 15)
Men who have sex with men	49 of 60	15 of 15
Age (median years with IQR)^A^	55 (47–61)	39 (35–42)
Plasma viral load		
≤50 copies/ml^B^	58 of 60	n.a.
>50 copies/ml	2 of 60	n.a.
CD4+ counts/μl		
Nadir (IQR) ^C^	235 (159–390)	n.a.
Actual (IQR)	606 (497–733)	n.a.
Years on ART (IQR)^D^	12 (8–21)	n.a.
Receiving ART	60 of 60	14 of 15 (HIV PrEP)
HPV vaccination status	1 of 60	1^E^ of 15
HPV associated anal abnormal cytology (including ASC-US, AIN1/LSIL, AIN2/HSIL)	17 of 54^F^	n.a.

^A^At baseline visit.

^B^≤50 copies/ml throughout all study visits.

^C^These data were unavailable from two individuals.

^D^One individual for whom these data were unavailable.

^E^HPV vaccination was performed after the baseline visit.

^F^Data were unavailable for six individuals. n.a.: not applicable.

**TABLE 2:** Overview of study visits.

	PLWH (*n* = 60)		PLWoH (*n* = 15)	
	Patients with available samples	Weeks between follow-up and baseline	Patients with available samples	Weeks between follow-up and baseline
Baseline visit	60 of 60	n.a.	15 of 15	n.a.
First follow-up	60 of 60	13 (IQR 13–15)	Not performed	n.a.
Second follow-up	56 of 60^A^	27 (IQR 25–28)	14 of 15	35 (IQR 31–45)
Third follow-up	56 of 60^A^	53 (IQR 51–55)	13 of 15	53 (IQR 52–56)

^A^One individual with missing second and third follow-up. n.a.: not applicable.

### Specimen processing

Blood sample processing was performed as described previously (Grüner et al., 2024; Ober et al., 2025). In brief, peripheral blood samples were collected in four potassium-EDTA-coated blood collection tubes (Sarstedt) and were processed within 2 h. Whole blood was centrifuged at 450 × *g* for 10 min at room temperature (RT). PBMCs were isolated by Histopaque 1077 (Sigma Aldrich) density gradient centrifugation. After counting (Casy 1TT; Schärfe System), PBMC were either freshly used or transferred to a cryo medium [90% FCS (Sigma Aldrich), 10% DMSO (Sigma Aldrich)] before cryopreserved in liquid nitrogen.

As described previously (Grüner et al., 2024), the collection of ano-mucosal specimens was performed with a moistened PAP-Cone (Menton Medizintechnik) that was inserted 2–3 cm into the anal canal, rotated with gentle pressure and then put into 3 ml ThinPrep*™* Preserve-Cyt solution (DNA extraction; Hologic) or in 5 ml R10 medium (cell isolation; RPMI-1640 (Sigma Aldrich) supplemented with 10% FCS, 1% L-glutamine, 1% Hepes, 1% penicillin/streptamycin). The tubes were vortexed immediately.

The PAP-Cone was removed from R10 media and washed with PBS over a 100 μm cell strainer (Greiner) to remove epithelial cells. Cells in the R10 media were pelleted, resuspended in PBS, filtered through the same cell strainer, transferred to cryo medium before being cryopreserved in liquid nitrogen.

### Anal HPV genotyping

For anal HPV genotyping, DNA was isolated from 500 μl ThinPrep™ Preserve-Cyt solution with QIAamp^®^ DNA Mini Kit (Qiagen; #51304) according to the manufacturer’s instruction. The multiplex real-time PCR assay (Allplex™ HPV28 Detection Kit; Seegene; #HP10372X) identifies low (LR: HPV6, 11, 40, 42, 43, 44, 54, 61), putative high risk (PHR including probably and possibly cancerogenic types: HPV26, 53, 66, 68, 69, 70, 73, 82) and high-risk HPV types (HR: HPV16, 18, 31, 33, 35, 39, 45, 51, 52, 56, 58, 59) as classified by WHO (National Cancer Institute, 2025). The manufacturer’s instructions were followed.

For the calculation of clearance/persistence proportions, three sampling time points per individual were considered to ensure comparability between groups. For men living with HIV who attended four visits, the sampling time points at baseline, week 27, and week 53 were included in the analysis. For individuals attending three visits, all available time points were considered. Individuals with fewer than three visits were excluded from the clearance/persistence analysis. Clearance was defined as absence of a baseline-detected type in two consecutive follow-ups. Persistence was defined as detection of a type at baseline, and two consecutive follow-up visits.

### IFN-γ ELISpot

ELISpot analysis was performed in individuals infected with at least one of the five high-risk HPV types (HPV16, 18, 35, 45, and 58), in whom one of these types was either cleared or persisted according to the definitions of clearance and persistence stated above. ELISpot analysis was conducted at two time points: baseline and 53-week follow-up.

To assess HR HPV-specific memory T cell responses, IFN-γ release from freshly isolated PBMC was measured after stimulation with 15mer HPV peptide pools (JPT Peptide Technologie) overlapping by 11 amino acids of oncoproteins E6 and E7 (HPV16, 18, 35, 45 and 58) and L1 and E2 (HPV16, 18 and 45) as previously described by Mbuya et al. (2021). For the assay, a 96-well filter plate (Merck Millipore; #MSIPN4W50) were first pre-wetted 4 times with 200 μl sterile PBS (Sigma-Aldrich) and then coated overnight with anti-human IFN-γ monoclonal antibody (Mabtech; 3420-3-250) in PBS (5 μg/ml). After removing any unbound antibody by washing the plate 3 times with sterile PBS, 200 μl R10 was added to each well for blocking (30 min, RT). Subsequently, the blocking solution was removed, and peptide pools were added in duplicates before seeding 200,000 freshly isolated PBMCs per well. HPV peptide pools were added with a final concentration of 2 μg/ml per peptide. HCMV pp65 (2 μg/ml; JPT Peptide Technologies), CERI-MHC-I (1:200; CTL), and PHA (2.5 μg/ml; Sigma Aldrich) served as positive control, while triplicates of R10-media wells only were used as negative control. Following O/N incubation (37°C, 5% CO_2_, 20 h), plates were washed with PBS and incubated with the biotinylated anti-IFN-γ monoclonal antibody in PBS (0.5 μg/ml; 2 h, dark, RT; 3420-6-250). After additional washing steps with PBS, streptavidin (1:2000 in PBS; Mabtech) was added (1 h, dark, RT). The plate was then developed using the AP Conjugate Substrate Kit (Biorad). After drying O/N (dark, RT), spots were counted using an automated ELISpot plate reader (AID ELISPOT reader ELR04; Autoimmun Diagnostika GmbH), which was manually controlled. The number of spot forming cells (SFC) was calculated by subtracting the mean of the negative controls (background signal). Responders were defined as ≥3x mean SFC of the negative controls and > 25 SFC/10^6^ PBMCs as described previously (Mbuya et al., 2021), and were required to be positive at both baseline and 1-year follow-up.

### Flow cytometry

For analysis of exhaustion markers on T cells, cryopreserved PBMCs were thawed, washed twice in R10 medium, and counted. 1 mio PBMCs were washed in PBS with 1% FCS and subsequently in PBS, before being stained for viability (15 min, dark, RT; Zombie NIR™ Fixable Viability Kit; BioLegend). Next, extracellular staining with fluorochrome-conjugated antibody was performed (20 min, dark, RT; Supplementary Table 2). Cells were washed in PBS with 1% FCS and fixed in Fix&Perm Medium A (15 min, RT; Thermo Fisher Scientific). After an additional wash step, cells were permeabilized with Fix&Perm Medium B (15 min, dark, RT; Thermo Fisher Scientific) and re-suspended in a mastermix containing antibodies for intracellular staining (Supplementary Table 2) and incubated for 30 min at RT. Cells were washed and resuspended in 200 μl PBS for acquisition on a BD LSR Fortessa (BD Bioscience). Fluorochrome compensation was performed using single-stained CompBeads (BD Bioscience; Supplementary Figure 1 for compensation matrix) and Rainbow beads (BD Bioscience) were run before every acquisition to ensure inter-experimental consistency. Data was analyzed with FlowJo software, version 10. The gating strategy is depicted in Supplementary Figure 2. Memory subsets were gated based on the CCR7 vs. CD45RA gate. TCF1^+^ and PD-1^+^ populations were gated FMO-guided. TCF1^–^PD-1^+^TIM3^+^ cells were detected using Boolean gates of TCF1^–^PD-1^+^ and TIM3^+^.

### Sorting of CD8^+^ T cells and low-input RNA sequencing

CD8^+^ T cells obtained from the anal swab of participants with or without HR HPV infection were sorted to analyze their transcriptional profile. Detailed clinical data on chosen donors are summarized in Supplementary Table 3. Frozen cells were thawed, washed in R10 with 0.2 μl/ml Benzonase (Novagen/Merck) followed by PBS and stained for viability with Zombie NIR™ Fixable Viability Kit and with fluorochrome-conjugated antibodies (Supplementary Table 2) simultaneously (20 min, dark, RT). After washing, cells were resuspended in FACS buffer (PBS containing 2 mM EDTA and 1% FCS) and filtered through a 35 μm cell strainer (Corning Science) and 10–50 CD8^+^ T cells were immediately sorted with a CytoFLEX SRT Benchtop Cell Sorter (Beckman Coulter Life Science) into single wells of a 96-well plate (Biozym) containing 5 μl lysis buffer (0.2% Triton-X-100; 100 μM Oligo dT primer, 10 mM dNTP mix, 40 U/μl recombinant RNase Inhibitor). The gating strategy and compensation matrix are depicted in Supplementary Figure 3. RNA isolation and library preparation was performed using modified version of the Smart-seq2 protocol (Picelli et al., 2014) and as described previously (Rueger et al., 2025). In brief, full length-RNA was reverse transcribed by SuperScript II Reverse Transcriptase (Thermo Fisher Scientific; #18064071) using oligo(dT) primers and a template switching primer, both containing additional sequences to prime the subsequent PCR amplification [0.2 μM universal primer; KAPA HiFi HotStart ReadyMix (Roche; #7958935001)]. Libraries were generated with Nextera XT DNA Library Preparation Kit (Illumina; #FC-131-1024) and Nextera XT Index Kit v2 Set (Illumina; FC-131-2001) according to manufacturer’s protocol but using one-quarter of the recommended volume. PCR products and libraries were purified using Agencourt AMPure XP beads (Beckman Coulter). DNA concentration was measured with Qubit dsDNA HS Assay Kit (Thermo Fisher Scientific; #Q32854) and size distribution was assessed using the Agilent High Sensitivity DNA Kit (Agilent; #5067–5593). 100 bp paired-end sequencing of the libraries was performed on Illumina NovaSeq X plus instrument with an average sequence depth of 1–4 × 10^6^ reads per cell.

Quality of raw sequencing reads was assessed using FastQC^1^. Sequence reads were mapped to the human genome reference GRCh38, v. 47 (release 113) using kallisto (v. 0.49) from bustools. *In silico* globin depletion and removal of mitochondrial genes was performed. Genes with less than 10 reads or those expressed in less than 30% of the patients across both groups were removed from the analysis. The resulting raw gene counts table served as input for DESeq2 (Love et al., 2014) with the design formula accounting only for HPV status. The poscounts option in Deseq2 was used to account for the low number of reads, noise and presence of dropouts. The remaining procedure followed the standard DESeq command using the default values, including the *p*-value adjustment by Benjamini-Hochberg method. Both shrinkage methods, ashr (Stephens, 2017) and apeglm (Zhu et al., 2019), were applied to verify that the genes were reported with an absolute log fold change (LFC) above threshold (LFC > 1) and reaching the significance level of 0.05 after FDR correction in both correction methods. Average library size per sample was 80 million reads, with average mapping rate of 0.71 (SD = 0.07). For details refer to Supplementary Figure 4 and Supplementary Table 4.

The R packages clusterProfiler (Yu et al., 2012) and ReactomePA (Yu and He, 2016) was used to investigate pathways enrichment among DEGs using Gene Ontology (GO) as well as KEGG and Reactome databases. Gene id conversions were performed using KEGGREST (Tenenbaum and Maintainer, 2025) and org.Hs.eg.db (Carlson, 2025) package version 3.22.0.

Additional packages used for data wrangling and visualization were: R: vroom and tidyverse (Wickham et al., 2019), ComplexHeatmap (Gu, 2022) and ggrepel (Slowikowski et al., 2024).

### Statistics

Statistical analyses were performed with GraphPad Prism version 10.1.2. Fisher’s exact- or Mann-Whitney-tests were used for two-group comparisons where applicable. *P*-values < 0.05 were considered statistically significant. Data are expressed as median with interquartile ranges, except where otherwise specified. During differential gene expression analysis Wald test was used to calculate *p*-values and Benjamini-Hochberg procedure to correct for multiple testing.

### Data availability

Low-input RNA sequencing data will be made available online in the European Nucleotide Archive (ENA) under the Accession Number: PRJEB110682. Raw data of flow cytometry and ELISPOT will be made available upon reasonable request to the corresponding author.

## Results

### High HPV prevalence in MSM independent of HIV infection

First, we assessed baseline HPV status in a cohort of 75 men, of whom 64 identified as men who have sex with men (MSM). Among MSM, 49/64 were living with HIV and 15/64 were HIV-negative. All 11 participants that defined themselves as non-MSM participants were living with HIV.

Men living with and without HIV differed in median age (55 vs. 39 years, respectively). However, given that anal HPV prevalence in men is less strongly age-dependent than genital HPV in women (Poynten et al., 2016), the study groups were considered comparable with respect to age distribution for the purposes of analysis.

**FIGURE 1:**
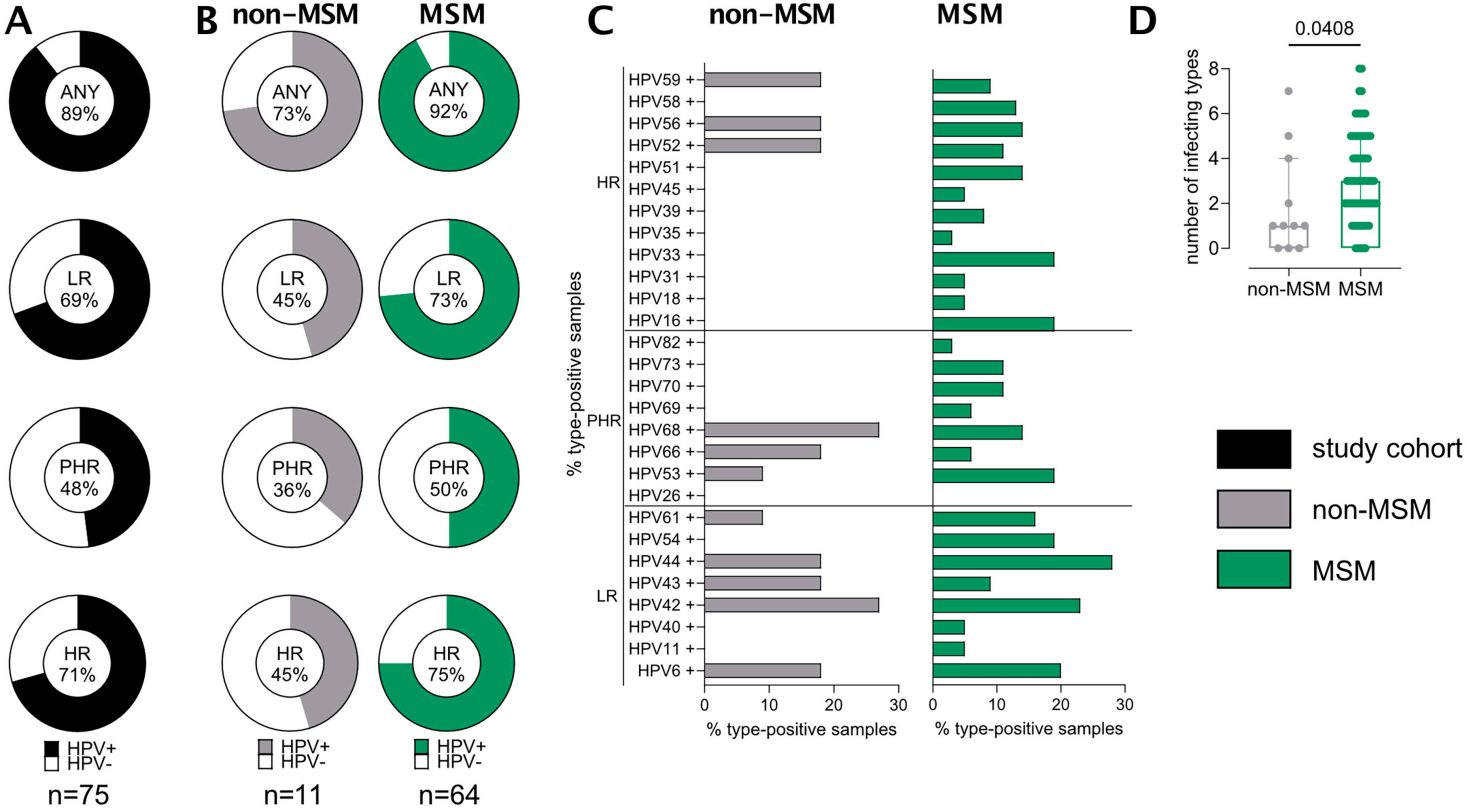
High HPV prevalence in MSM independent of HIV infection. **(A)** Donut plots showing the percentage of individuals (*n* = 75) infected with any of the 28 tested HPV types (ANY) (67/75), low-risk (LR) (52/75), probably and possibly cancerogenic types (PHR) (36/75), or high-risk (HR) HPV (53/75) types at baseline. **(B)** Same as in panel **(A)**, shown separately for men identifying as non-MSM (left, gray, *n* = 11) and MSM (right, green, *n* = 64). **(C)** Bar plots showing the percentage of all individuals positive for different HPV types in MSM (green) and non-MSM (gray). **(D)** Number of infecting HPV types per individual in non-MSM (gray) compared to MSM (green). Statistical significance was assessed using a Mann–Whitney test with an α level of 0.05. **(A–D)** Anal HPV status at baseline was assessed with Allplex™ HPV28 Detection Kit.

The overall burden of anal HPV infection was high, with 89% (67/75) of participants testing positive for at least one HPV genotype at baseline (Figure 1A). When stratifying HPV genotypes by oncogenic risk (National Cancer Institute, 2025) we again observed a substantial burden of HR HPV infection, with 71% (53/75) of participants testing positive for at least one HR HPV genotype (Figure 1A).

The prevalence of any HPV infection was higher in MSM (living with and without HIV) than non-MSM living with HIV [92% (59/64) vs. 73% (8/11)]. A similar trend was observed for HR HPV infection, with 75% (48/64) in MSM compared with 45% (5/11) in non-MSM (Figures 1B,C). These findings did not reach statistically significance, likely due to the unequal group sizes.

To assess differences in HPV infection between men living with HIV and men living without HIV we stratified the cohort by HIV status. All men living with HIV in this cohort were receiving ART. 58 of 60 had undetectable plasma viral loads throughout the whole study, and the median CD4^+^ T cell count was 606/μL (Table 1 and Supplementary Table 1 for detailed HIV-associated clinical characteristics at patient level). 14/15 men without HIV were receiving pre-exposure prophylaxis (PrEP). The overall anal HPV prevalence was 93% (14/15) among MSM living without and 88% (53/60) among men living with HIV (Supplementary Figure 5A). Among men living with HIV, 92% of MSM (45/49) were positive for at least one HPV type, compared with only 73% (8/11) of non-MSM. In addition, a significantly higher number of distinct HPV types could be detected at baseline (median = 3) in MSM compared with non-MSM (median = 1) (Figure 1D). This difference was not observed when participants were stratified by HIV status (Supplementary Figure 5B).

### Clearance of HR HPV infection is associated with HPV type-specific T cell response

Given an overall high HPV burden in our cohort, we next assessed HPV clearance and persistence dynamics within the 1-year follow-up. In total, 167 infections that fall into our clearance/persistence definition were detected, of which 68 classified as LR HPV, 34 as PHR HPV and 65 as HR HPV.

**FIGURE 2:**
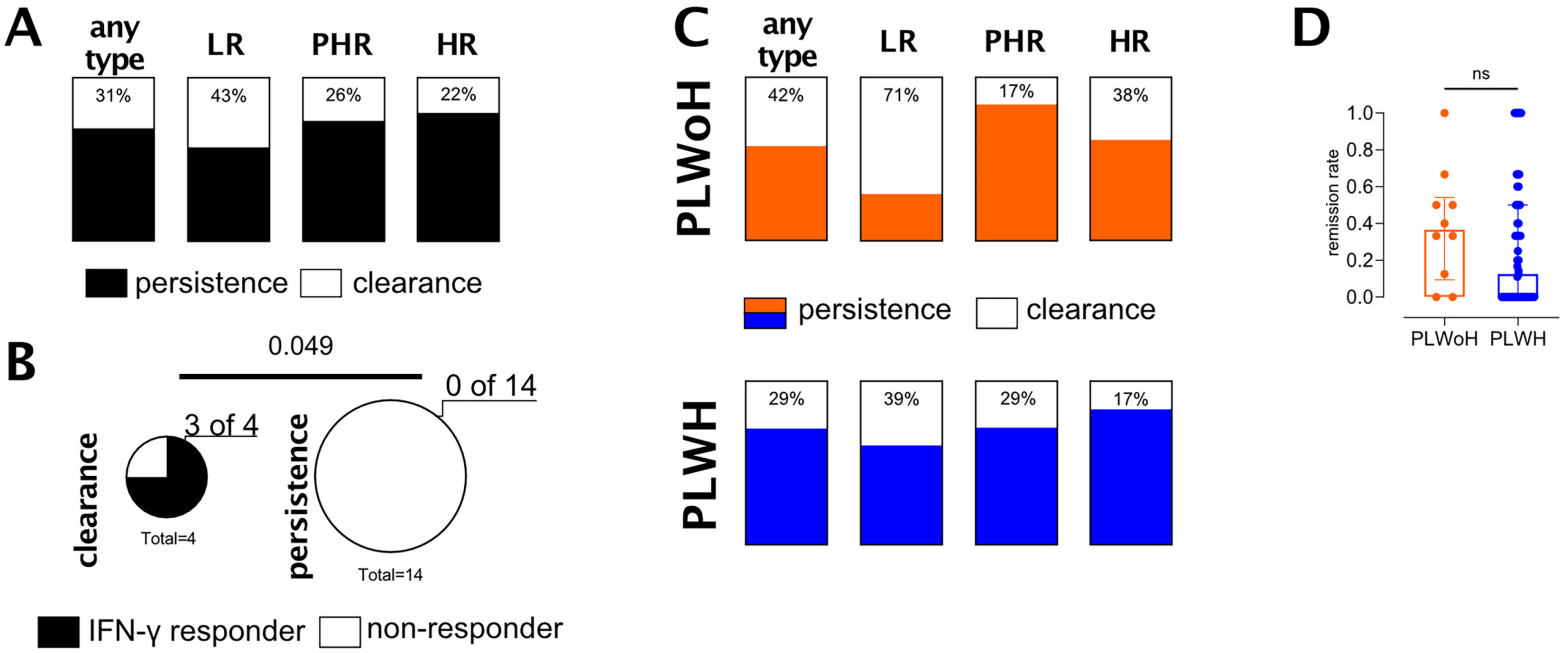
Clearance of HR HPV infection is associated with type-specific T cell response. **(A)** Rectangles indicate the proportions of all, low-risk (LR) (*n* = 68), probably and possibly cancerogenic types (PHR) (*n* = 34), and high-risk (HR) (*n* = 65) HPV infections that were either cleared (white) or remained persistent (black) at a type-specific level within the 1-year follow-up period. The percentage of cleared infections is shown within the white squares. Clearance was defined as absence of a baseline-detected type in two consecutive follow-ups. Persistence was defined as detection of a type at baseline, and two consecutive follow-up visits. Intermediate patterns were not classified as either clearance or persistence and were not taken into account. One individual living with and three individuals living without HIV were excluded from this analysis as less than three study visits were available. **(B)** Individuals were stratified according to clearance or persistence of one of the high-risk (HR) HPV genotypes 16, 18, 35, 45, or 58. Pie charts depict the proportion of individuals within each group exhibiting a positive IFN-γ response at baseline and 1 year follow up (responders) to the corresponding HPV genotype that was either cleared or persisted. Pie charts represent cohort sizes (clearance: *n* = 4; persistence: *n* = 14) and are scaled by area of the circle. Statistical significance was assessed using Fisher’s exact test with an α level of 0.05; *P*-value is indicated above the bar graphs. **(C)** Same as in panel **(A)**, stratified by HIV status: men living without HIV (orange, top) or with HIV (blue, bottom). **(D)** Clearance rate of any anal HPV type detected at baseline visit calculated as number of cleared types divided through number of all detected HPV types at baseline and stratified by HIV status (Median 0.4 for men living without HIV and 0.1 for men living with HIV). Participants negative for anal HPV infection at baseline are excluded from this analysis. ns – non significant.

Within our cohort, the majority of anal HPV infections were persistent, with only 31% (52/167 infections) clearance during the 1-year observation period. Clearance was more common among LR HPV genotypes (43%, 29/68 infections), whereas HR genotypes showed a marked tendency to persist, with only 22% (14/65 infections) clearing within 1 year (Figure 2A).

To elucidate potential mechanisms contributing to HPV clearance or persistence, we examined peripheral T cell responses to genotype-specific HPV antigens (E6, E7, and L1) from HR HPV types 16, 18, 35, 45, and 58 by IFN-γ ELISpot. Responder rates to HR HPV types were few and ranged from 0% to 33%, depending on the respective genotype, with no differences observed between individuals living with and without HIV (Supplementary Figures 6A,B). To assess the relationship between genotype-specific T cell responses and clearance or persistence, we next analyzed type-specific T cell responses directed against HPV genotypes that were either cleared or persisted within the same individual.

We detected systemic T-cell responses against the corresponding type-specific antigen in 3 of 4 individuals (75%) who had cleared the respective HPV genotype. In contrast, among individuals with persistence of a given HPV type, none of 14 demonstrated a systemic antigen-specific response (Figure 2B, Fisher’s exact test, *p* = 0.049). These findings point toward a potential association between HPV clearance and the presence of virus-specific, systemic T-cell responses.

To further explore factors that might influence HPV clearance, we stratified outcomes by HIV status and observed a trend toward reduced overall HPV clearance among people living with HIV. This was seen for both LR genotypes [39% (24/61 infections) vs. 71% (5/7 infections)] and HR genotypes [17% (9/52 infections) vs. 38% (5/13 infections)] (Figure 2C). Having calculated a per-person clearance rate for any HPV type, we see a similar trend with a median clearance rate of 0.4 in men living without HIV and 0.1 in men living with HIV (Figure 2D). These findings, however, did not reach statistical significance likely due to unequal size of comparison groups.

### Markers of activation, exhaustion and senescence are increased on bulk CD4^+^ and CD8^+^ T cells in men living with HIV and infected with at least one HR HPV strain

**FIGURE 3:**
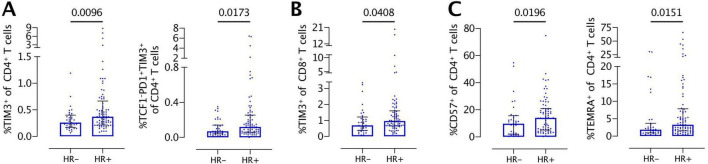
Markers of activation, exhaustion and senescence are increased on peripheral T cells in men living with HIV with HR HPV infection. **(A,B)** Frequency of CD4^+^ T cells **(A)** or CD8^+^ T cells **(B)** expressing exhaustion markers in men living with HIV with or without at least one HR HPV type. **(C)** Frequency of CD4^+^ T cells expressing senescence markers in men living with HIV with or without at least one HR HPV type. **(A–C)** Each individual is represented by two data points corresponding to baseline and 1-year follow-up sampling (week 53). All graphs display the median with interquartile range. Statistical significance was determined using the Mann–Whitney test with an α level of 0.05. *P*-values are indicated above the bar graphs. One individual with no 1-year follow-up was excluded from this analysis.

Given the trend toward lower HPV clearance in men living with HIV, we next examined whether specific immunological alterations are associated with HR HPV status in this population. We analyzed peripheral T cells for markers of exhaustion and senescence at two time points (baseline and 1-year follow-up) in 59 of 60 men living with HIV, grouped according to HR HPV infection status. One individual without 1-year follow-up data had to be excluded from this analysis. Study participants were considered HR HPV positive if they carried at least one HR HPV strain at the respective sampling time point. We found the exhaustion marker Tim-3 to be significantly enriched on peripheral CD4^+^ and CD8^+^ T cells in men living with HIV and positive for at least one HR HPV type (Figures 3A,B). In addition, a subset of TCF1^–^ PD-1^+^ TIM-3^+^ CD4^+^ T cells was increased in men living with HIV with HR HPV infection (Figure 3A).

Alongside these features of exhaustion, we also observed increased expression of the senescence marker CD57 on CD4^+^ T cells in men living with HIV with HR HPV infection, as well as an enrichment of terminally differentiated T cells re-expressing CD45RA (TEMRA; Figure 3C).

Expression of CTLA-4, PD-1, and BTLA on both CD4^+^ and CD8^+^ T cells showed a modest upward trend in the presence of HR HPV infection, although these differences did not reach statistical significance (Supplementary Figure 7).

Taken together, our data indicate a modest increase in peripheral T cell exhaustion and senescence in men living with HIV and anal HR HPV infection compared to those without HR HPV infection.

### Upregulation of genes associated with immune activation and effector functions but also exhaustion in anal tissue-resident bulk CD8^+^ T cells of individuals with HR HPV infection

We next focused on alterations in anal tissue-resident immune cells, which are thought to significantly contribute to HPV clearance or persistence (Guo et al., 2023; Ntuli et al., 2022). CD8^+^ T cells are believed to play a critical role in this context, as they have been described in anogenital warts and in cervical intraepithelial neoplasia (CIN), where their presence correlates with lesion regression (McKenzie et al., 1991; Shen et al., 2022; Coleman et al., 1994).

In our cohort, we performed low-input RNA sequencing on sorted CD8^+^ T cells from ano-mucosal tissue of six donors: three in whom at least one HR HPV type was detected throughout all time points, and three in whom no HPV infection was observed at the sampled time point. Detailed information on anal HPV status und HIV related data is shown in Supplementary Table 3 for the six donors. For each donor with HR HPV, the time point yielding the highest number of sorted cells was selected. All six donors were living with HIV.

We first observed that in individuals with HR HPV infection genes associated with a tissue-resident memory phenotype (*CAPG*; log_2_FC = 4.0, padj = 0.03) and intraepithelial lymphocytes (*KIR2DL4*; log_2_FC = 6.6, padj < 0.01) were upregulated. The lymphoid homing marker *SELL* was downregulated (log_2_FC = −4.3, padj = 0.04), suggesting retention of CD8^+^ T cells at the local site.

**FIGURE 4:**
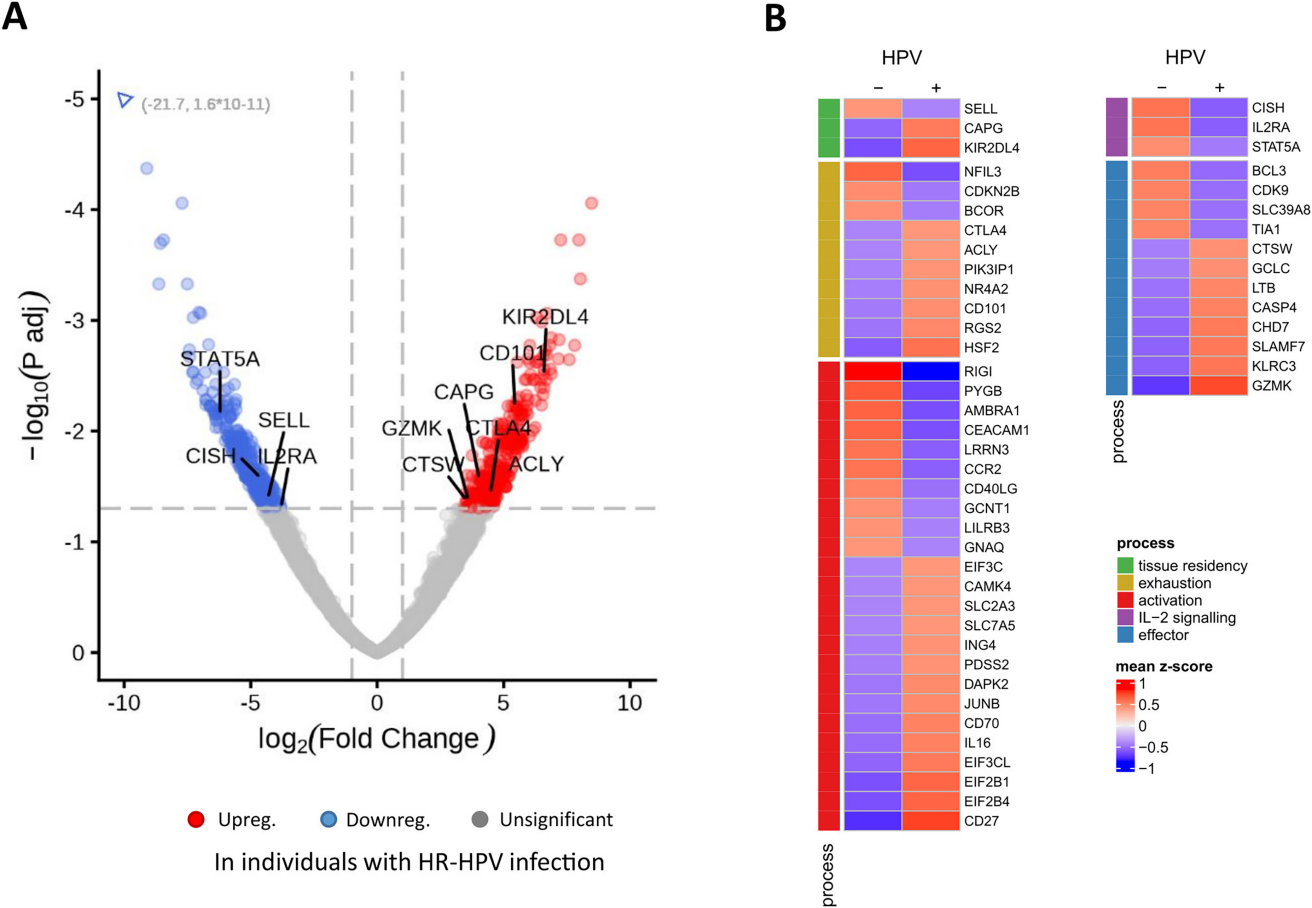
Upregulation of genes associated with immune activation and effector functions but also exhaustion in anal CD8^+^ T cells of individuals with HR HPV infection. **(A)** Volcano plot depicting upregulated (red) and downregulated (blue) DEGs in anal CD8^+^ T cells from individuals with HR HPV infection compared to those without HR HPV infection at the sampling time point. Cutoffs for differential expression: adjusted *P*-value < 0.05 and absolute log_2_ (fold-change) > 1 (N_*tested*_ = 13253, N_*sig.*_ = 798). Selected immune-related genes are highlighted. **(B)** Average expression of selected DEGs in anal CD8^+^ T cells in the group of individuals with HR HPV infection versus anal CD8^+^ T cells from the group without HR HPV infection categorized according to function. Relative changes in gene expression between CD8^+^ T cells from individuals with HR HPV infection and those without HR HPV infection are depicted. Normalized read counts, calculated with DESeq2, were converted into z scores and mean z scores per group for each gene are plotted. Plotted DEGs were selected based on their function. Red represents higher and blue lower gene expression.

Consistent with our observations on systemic T cells, our data shows features of exhaustion in CD8^+^ T cells residing at the ano-mucsal site from the three donors with HR HPV. Transcripts for exhaustion markers *CTLA4* (log_2_FC = 4.5, padj = 0.04) and *CD101* (log_2_FC = 5.4, padj = 0.01), which are indicative of terminally exhausted CD8^+^ T cells, were upregulated (Figures 4A,B). Also, *ATP citrate lyase* (*ACLY*, log_2_FC = 4.6, padj = 0.03), an enzyme involved in metabolic reprogramming that enforces differentiation toward an exhausted CD8^+^ T cell phenotype (Ma et al., 2025), was upregulated in the context of HR HPV infection.

In contrast to the consistent upregulation of exhaustion-associated genes, genes linked to activation exhibited a more balanced pattern of up- and downregulation (Figures 4A,B). This is not unexpected, as we analyzed bulk CD8^+^ T cells sampled at the anal mucosa and did not focus on a defined subset. Interestingly, key markers of IL-2 signaling [*IL2RA* (log_2_FC = −3.8, padj = 0.05), *STAT5A* (log_2_FC = −6.2, padj = 0.01), and *CISH* (log_2_FC = −4.6, padj = 0.03)] were all downregulated in CD8^+^ T cells from the anal mucosa of HR HPV–positive men, suggesting reduced IL-2 signaling.

We observed upregulation of genes associated with cytotoxic effector function, including *granzyme K* (*GZMK*; log_2_FC = 3.6, padj = 0.04) and *cathepsin W* (*CTSW;* log_2_FC = 3.5, padj = 0.04), in CD8^+^ T cells at sites of HR HPV infection. However, whether cytotoxic effector function is truly preserved requires validation in functional cytotoxicity assays.

Taken together, low-input RNA sequencing of CD8^+^ T cells in the ano-mucosal region of HR HPV infected individuals points toward a heterogeneous T cell landscape. It is characterized by features of exhaustion supporting our findings on systemic T cells, alongside activation and presumably cytotoxic effector function.

## Discussion

In this study, we characterized HPV prevalence and HPV-associated risk factors in a cohort of 75 men, including 60 men living with HIV, of whom 64 self-identified as MSM. In a subgroup, we additionally investigated immunological mechanisms of HPV infection in men living with HIV.

Overall, HPV prevalence was high, both among MSM and non-MSM, and was independent of HIV status. At baseline, most participants harbored at least one HPV infection, with 71% positive for at least one HR HPV type. Most studies report a higher HPV prevalence among MSM living with HIV than among MSM living without HIV (Critchlow et al., 1998; Palefsky et al., 1990; Palefsky et al., 1998; Bai et al., 2024; Zhou et al., 2021). This pattern, however, was not observed in our cohort where MSM living without HIV exhibited a slightly higher HPV burden at baseline (93%, 14/15) than men living with HIV (88%, 53/60) without significant difference in the number of infecting HPV types. Among men living with HIV, 92% of MSM (45/49) and 73% of non-MSM (8/11) tested positive for at least one HPV type. We hypothesize that this finding may reflect the unique characteristics of our cohort, since almost all men without HIV are using PrEP (14/15) and not all men living with HIV were MSM. Thus, sexual risk behavior and risk compensation associated with PrEP use may increase the prevalence of reported sexually transmitted infections, potentially attenuating the expected effect of HIV infection on anal HPV rates (Rossotti et al., 2022; Ucciferri et al., 2018; Traeger et al., 2019).

Clearance proportion of any HPV infection (31%) including HR HPV infections (22%), observed in our cohort over the 1-year follow-up period were low relative to previous reports, which describe clearance of approximately 50%–75% of baseline HPV infections overall and more than 50% of HR HPV infections (Nyitray et al., 2011; van Aar et al., 2014; Taylor et al., 2016). These discrepancies may be explained by our stringent definition of clearance: a baseline HPV type was considered cleared only if undetectable at two consecutive follow-up visits. Consequently, re-infections with the same HPV genotype cannot be distinguished from persistent infections that may have fallen below the limit of detection at intermittent visits due to sampling variability.

Given the high proportion of persistent HPV infections, understanding the factors that influence clearance becomes particularly important. Our data, although restricted by numbers, indicate that type-specific T-cell responses could be associated with clearance of the corresponding HR HPV type. These findings are supported by T-cell responses in women with cervical HPV infection that have been detected only after recent clearance, but not in persistent infection (Farhat et al., 2009; Nakagawa et al., 2000). Taken together, our findings, along with previous reports, support an association of systemic T-cell responses with HPV clearance.

HIV infection status is a well-known risk factor significantly influencing HPV clearance (Critchlow et al., 1998; de Pokomandy et al., 2009; Wei et al., 2023). Our data shows a clearance proportion of any baseline HPV infection in 29% of men living with HIV compared to 38% of men living without HIV. This difference was even more pronounced for HR HPV infections (17% vs. 38%). Reduced clearance of HPV infections in people living with HIV has been consistently reported, with some studies describing approximately halved clearance rates (Looker et al., 2018; Mbulawa et al., 2012; Breese et al., 1995). Similar findings were reported for oral HPV16 in MSM living with vs. without HIV (van Aar et al., 2014). However, the mechanisms underlying remain unclear, particularly in our cohort of men living with stable HIV infection under suppressive ART, with median CD4^+^ counts >600 counts/μl, and without overt signs of advanced HIV-associated immunodeficiency. Persistent immune activation and functional immune dysregulation despite effective ART have been well described, including in the context of chronic viral co-infection (Kottilil et al., 2009; Kovacs et al., 2008; Freeman et al., 2016). Consistent with this concept, our analysis of peripheral T cells from men living with HIV with current HR HPV infection show slightly increased expression of markers associated with T-cell exhaustion and senescence.

Features of T-cell exhaustion in peripheral blood linked to cervical HR HPV infection has also been reported in women living with HIV, notably independent of the presence of cervical intraepithelial neoplasia (Papasavvas et al., 2016). In conclusion, our findings, together with previous reports, suggest that HR HPV infection in men living with HIV may be associated with features consistent with exhaustion in peripheral CD4^+^ and CD8^+^ T cells.

The local immune landscape at the site of HPV infection is known to play a critical role in viral control and disease progression. In the context of cervical neoplasia, it has been shown that progression from normal cervical epithelium through HPV infection and high-grade squamous intraepithelial lesions to cervical cancer is accompanied by marked changes in CD4^+^ T-cell responses. These include a shift from an initial antiviral Th1-dominated response toward Th17/Treg and Th1/Treg imbalances. This shift is thought to impair effective HPV clearance and ultimately promote carcinogenesis (Guo et al., 2023). In parallel, CD8^+^ T cells, which initially exhibit cytotoxic effector functions, progressively decline during carcinogenesis and adopt an increasingly exhausted phenotype (Guo et al., 2023).

We compared the immune profile of ano-mucosal CD8^+^ T cells from 3 individuals with a persistent anal infection with at least one HR HPV type with 3 HR HPV negative individuals.

Our results reveal a heterogeneous immune landscape in HR HPV infection, most likely due to the simultaneous presence of distinct CD8^+^ T cell subsets. Ano-mucosal CD8^+^ T cells exhibit features of exhaustion supporting our findings on systemic T cells, but also activation and likely preserved cytotoxic effector function. The coexistence of antivirally active and exhausted CD8^+^ T cells has also been observed in malignancies (Eberhardt et al., 2021; Li et al., 2022). For example, single-cell RNA-sequencing of tetramer-sorted HPV-specific and tumor-infiltrating CD8^+^ T cells in HNSCC identified both, effector-like subsets (expressing *NR4A1*, *FOS*, and *JUN*) and exhausted populations marked by expression of *CTLA4, TOX, PDCD1*, and *TIGIT* (Eberhardt et al., 2021). Moreover, our data may indicate downregulation of IL-2 signaling in mucosal CD8^+^ T cells in persistently HR HPV-infected tissue. These findings are in agreement with previous reports suggesting that a diminished or impaired antiviral Th1–dominated immune response is associated with HPV persistence and the subsequent development of high-grade dysplasia (Guo et al., 2023; Clerici et al., 1997; Zhu et al., 2023).

Human papillomavirus infection outcome might therefore be influenced by a delicate balance between immune activation and effective antiviral effector function, which gradually shifts toward immune imbalance and exhaustion during disease progression (Guo et al., 2023). Our findings raise the possibility that early alterations in this balance may already be present at the stage of HR HPV infection. However, our data do not allow conclusions on whether persistent HPV infection contributes to an exhausted immune phenotype through chronic immune activation, or whether stronger immune exhaustion in some individuals living with HIV may facilitate HPV persistence.

Our study has several limitations. The relatively small sample size (*n* = 75) and the uneven comparison groups (*n* = 64 MSM vs. *n* = 11 non-MSM; and *n* = 60 men living with HIV vs. *n* = 15 men living without HIV) substantially limit the interpretation and statistical power of our analyses. This imbalance reflects the sampling process and the epidemiology of HIV in Germany, where HIV predominantly affects MSM.

Clearance was defined as the loss of a baseline-detected HPV type at two consecutive follow-up visits. We intentionally chose a stringent definition of clearance to minimize the risk of interpreting transient non-detection as true viral clearance. However, we acknowledge that this approach may fail to capture early clearance events or distinguish clearance followed by reinfection. In addition, the study design only allowed evaluation of clearance or persistence at the final follow-up approximately 1 year after baseline. As a consequence, calculation of time-to-clearance was not feasible due to the limited temporal resolution of the data, allowing only qualitative interpretation of clearance dynamics.

Importantly, HR HPV clearance of the types analyzed with the ELISpot was a rare event in our cohort (*n* = 4). Although our data suggests an association between HR HPV clearance and type-specific immune responses, the results must be interpreted with caution due to the limited sample size. Validation in larger cohorts will be required.

Furthermore, results from low-input RNA sequencing of ano-mucosal CD8^+^ T cells should be considered exploratory as performed in only six individuals.

Taken together, our findings support an association between HR HPV infection and immune dysfunction in men. Clinically, these observations may help identify individuals at increased risk of persistent infection. However, larger prospective studies with serial mucosal immune profiling will be required to validate these findings and to better understand the mechanisms underlying immune dysfunction in individuals infected with HR HPV.

## Data Availability

Low-input RNA sequencing data is available online in the European Nucleotide Archive (ENA) under the Accession Number: PRJEB110682.

## Footnotes

1
https://www.bioinformatics.babraham.ac.uk/projects/fastqc/
