# Supplementary material for: Local and systemic immune correlates of anal high-risk HPV infection and clearance in men living with HIV and men at high risk for HIV
Source: Front Microbiol. 2026 Jun 25;17:1855627. doi: 10.3389/fmicb.2026.1855627 (PMC13346060; doi:10.3389/fmicb.2026.1855627)
Supplement: Supplementary file 1 [file Data_Sheet_1.docx]

**Supplementary Table 1: HIV-associated clinical characteristics at a patient level.**

| Nr | CDC clinical category^A^ | CDC immunological category^B^ | CD4 nadir (counts/µl) | current CD4 counts (counts/µl) | years on ART^c^ | plasma HIV viral load >50copies/ml during study period^D^ |
| --- | --- | --- | --- | --- | --- | --- |
| 01 | C | 3 | 120 | 476 | 2 | always <50cp/ml |
| 02 | A | 2 | 359 | 707 | 10 | always <50cp/ml |
| 03 | A | 3 | 168 | 516 | 20 | always <50cp/ml |
| 04 | B | 3 | 199 | 734 | 18 | always <50cp/ml |
| 05 | B | 3 | 123 | 689 | 21 | always <50cp/ml |
| 06 | A | 2 | 241 | 871 | 33 | always <50cp/ml |
| 07 | C | 3 | missing data | 506 | missing data | always <50cp/ml |
| 08 | B | 3 | missing data | 323 | 27 | always <50cp/ml |
| 09 | A | 2 | 465 | 1109 | 7 | always <50cp/ml |
| 10 | A | 2 | 376 | 630 | 5 | always <50cp/ml |
| 11 | A | 2 | 256 | 506 | 3 | always <50cp/ml |
| 12 | A | 2 | 210 | 533 | 8 | always <50cp/ml |
| 13 | A | 2 | 370 | 595 | 6 | always <50cp/ml |
| 14 | A | 2 | 468 | 877 | 27 | >200,000cp/ml |
| 15 | C | 3 | 122 | 822 | 18 | always <50cp/ml |
| 16 | A | 2 | 430 | 628 | 7 | always <50cp/ml |
| 17 | B | 2 | 409 | 863 | 26 | always <50cp/ml |
| 18 | B | 3 | 174 | 533 | 18 | always <50cp/ml |
| 19 | B | 3 | 31 | 486 | 6 | always <50cp/ml |
| 20 | B | 2 | 274 | 518 | 11 | always <50cp/ml |
| 21 | A | 3 | 103 | 488 | 10 | always <50cp/ml |
| 22 | A | 2 | 236 | 619 | 14 | always <50cp/ml |
| 23 | B | 2 | 457 | 1620 | 10 | always <50cp/ml |
| 24 | C | 1 | 565 | 557 | 4 | always <50cp/ml |
| 25 | A | 2 | 316 | 993 | 21 | always <50cp/ml |
| 26 | B | 3 | 158 | 302 | 0 | always <50cp/ml |
| 27 | A | 1 | 509 | 628 | 1 | always <50cp/ml |
| 28 | A | 1 | 517 | 572 | 12 | always <50cp/ml |
| 29 | B | 2 | 455 | 683 | 7 | always <50cp/ml |
| 30 | A | 2 | 230 | 644 | 11 | always <50cp/ml |
| 31 | A | 2 | 246 | 566 | 13 | always <50cp/ml |
| 32 | C | 3 | 60 | 512 | 10 | BLIP at first and second follow-up visit |
| 33 | C | 3 | 4 | 721 | 26 | always <50cp/ml |
| 34 | A | 3 | 170 | 376 | 26 | always <50cp/ml |
| 35 | A | 1 | 617 | 732 | 20 | always <50cp/ml |
| 36 | C | 1 | 521 | 884 | 23 | always <50cp/ml |
| 37 | A | 3 | 169 | 738 | 17 | always <50cp/ml |
| 38 | A | 3 | 160 | 284 | 11 | always <50cp/ml |
| 39 | B | 3 | 139 | 573 | 10 | always <50cp/ml |
| 40 | B | 2 | 394 | 524 | 25 | always <50cp/ml |
| 41 | C | 3 | 40 | 337 | 19 | always <50cp/ml |
| 42 | B | 3 | 20 | 183 | 6 | always <50cp/ml |
| 43 | C | 3 | 10 | 155 | 2 | always <50cp/ml |
| 44 | C | 3 | 54 | 491 | 11 | always <50cp/ml |
| 45 | A | 2 | 202 | 755 | 11 | always <50cp/ml |
| 46 | A | 1 | 601 | 1220 | 12 | always <50cp/ml |
| 47 | A | 2 | 271 | 1093 | 21 | always <50cp/ml |
| 48 | A | 2 | 412 | 898 | 11 | always <50cp/ml |
| 49 | B | 2 | 241 | 707 | 14 | always <50cp/ml |
| 50 | C | 3 | 54 | 497 | 23 | always <50cp/ml |
| 51 | missing data | missing data | 260 | 409 | 0 | always <50cp/ml |
| 52 | C | 3 | 190 | 594 | 12 | always <50cp/ml |
| 53 | A | 2 | 303 | 637 | 25 | always <50cp/ml |
| 54 | A | 2 | 350 | 679 | 23 | always <50cp/ml |
| 55 | B | 3 | 170 | 616 | 8 | always <50cp/ml |
| 56 | B | 3 | 193 | 469 | 14 | always <50cp/ml |
| 57 | A | 2 | 234 | 497 | 6 | always <50cp/ml |
| 58 | C | 3 | 4 | 276 | 21 | always <50cp/ml |
| 59 | B | 1 | 564 | 1153 | 12 | always <50cp/ml |
| 60 | B | 3 | 190 | 639 | 24 | always <50cp/ml |

^A^ CDC clinical category (1993 classification) with A: asymptomatic HIV infection, persistent generalized lymphadenopathy or acute HIV illness; B: history of HIV-associated illness; C: history of AIDS-defining illness
^B^ CDC immunological category (1993 classification) with 1: CD4-nadir ≥500cell/µl; 2: CD4-nadir between 200 and 499 cells/µl; 3: CD4-nadir <200 cells/µl
^c^ ART: anti-retroviral therapy;
^D^ cp/ml: copies per ml

**Supplementary Table 2: List of antibodies used for flow cytometry of markers of exhaustion and sorting of ano-mucosal CD8 T cells.**

| **Antigen** | **Fluoro-chrome** | **Clone** | **Source** | **Identifier** | **Volume per Sample** | **Staining** | **Flow cytometry** | **Sorting** |
| --- | --- | --- | --- | --- | --- | --- | --- | --- |
| CD4 | BUV395 | RPA-T4 | BD Bioscience | 564724 | 0.5µl | ECS | x |  |
| CD3 | BUV496 | UCHT1 | BD Bioscience | 612940 | 0.5µl | ECS | x |  |
| CD39 | BUV737 | TU66 | BD Bioscience | 612852 | 1µl | ECS | x |  |
| PD-1 | BV421 | NAT105 | BioLegend | 367422 | 1µl | ECS | x |  |
| CD8 | BV480 | RPA-T8 | BD Bioscience | 566121 | 0.5µl | ECS | x |  |
| CXCR5 | BV650 | RF8B2 | BD Bioscience | 740528 | 1µl | ECS | x |  |
| CD25 | BV711 | 2A3 | BD Bioscience | 563159 | 1µl | ECS | x |  |
| BTLA | BV785 | J168-540 | BD Bioscience | 743988 | 3µl | ECS | x |  |
| CD45RA | BB515 | HI100 | BD Bioscience | 564552 | 2µl | ECS | x |  |
| TIM-3 | PE | F38-2E2 | BioLegend | 345006 | 5µl | ECS | x |  |
| CCR7 | PE-Dazzle | G043H7 | BioLegend | 353236 | 2µl | ECS | x |  |
| CD57 | PE-Cy7 | HNK-1 | BioLegend | 359624 | 2µl | ECS | x |  |
| CD127 | APC | A019D5 | BioLegend | 351316 | 2µl | ECS | x |  |
| CTLA-4 | PE-Cy5 | BNI3 | BD Bioscience | 555854 | 5µl | ICS | x |  |
| TCF-1 | R718 | S33-966 | BD Bioscience | 567587 | 5µl | ICS | x |  |
| CD4 | PE | RPA-T4 | BioLegend | 300508 | 1µl | ECS |  | x |
| CD45 | AF700 | 2D1 | BioLegend | 368513 | 2µl | ECS |  | x |
| CD3 | BV421 | Okt 03 | BioLegend | 317343 | 1µl | ECS |  | x |
| CD8 | BV510 | SK1 | BioLegend | 344732 | 1µl | ECS |  | x |

**Supplementary Table 3: Characteristics of patients included in low-input RNA-seq.**

| Nr. | Anal HPV status | Age (years) | CD4 nadir (cells/µl) | CD4 cell count (cells/µl) | Years on ART | HIV plasma viral load (cp/ml) |
| --- | --- | --- | --- | --- | --- | --- |
| 14 | no HPV infection at analyzed visit | 54 | 468 | 877 | 27 | <50 |
| 34 | no HPV infection at analyzed visit | 57 | 170 | 376 | 26 | <50 |
| 58 | no HPV infection at analyzed visit | 58 | 4 | 276 | 21 | <50 |
| 12 | HPV33, HPV51 infection throughout all visits (amongst others) | 37 | 210 | 533 | 8 | <50 |
| 16 | HPV33, HPV35, HPV53 infection throughout all visits (amongst others) | 42 | 430 | 628 | 7 | <50 |
| 21 | HPV39 infection throughout all visits (amongst others) | 39 | 103 | 488 | 10 | <50 |

**Supplementary Table 4: Quality-control metrics of RNA sequencing analysis**

|  | Group | Raw reads | Aligned Reads | % aligned |
| --- | --- | --- | --- | --- |
| 12 | HPV positive | 62746695 | 47932737 | 76.4 |
| 16 | HPV positive | 68160303 | 51882010 | 76.1 |
| 21 | HPV positive | 66564503 | 47454823 | 71.3 |
| 34 | HPV negative | 189081174 | 150254734 | 79.5 |
| 14 | HPV negative | 56726623 | 35458379 | 62.5 |
| 58 | HPV negative | 47584559 | 29377253 | 61.7 |

**
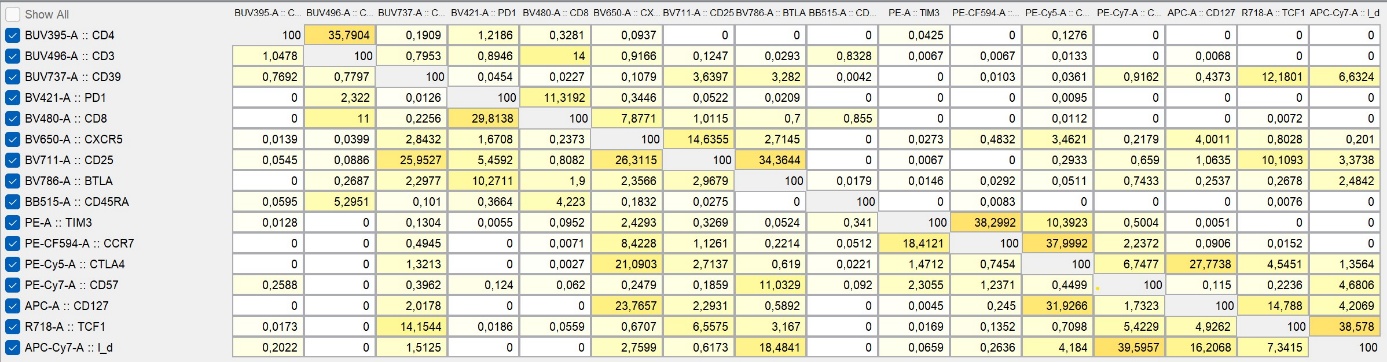
**

**
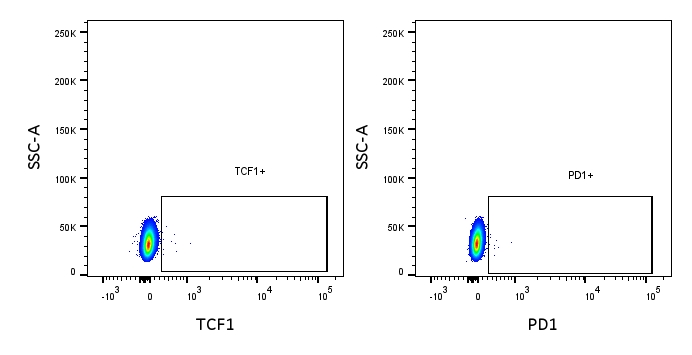
**

**Supplementary Figure 1: Compensation matrix and FMO controls for flow cytometry of exhaustion markers.**

**
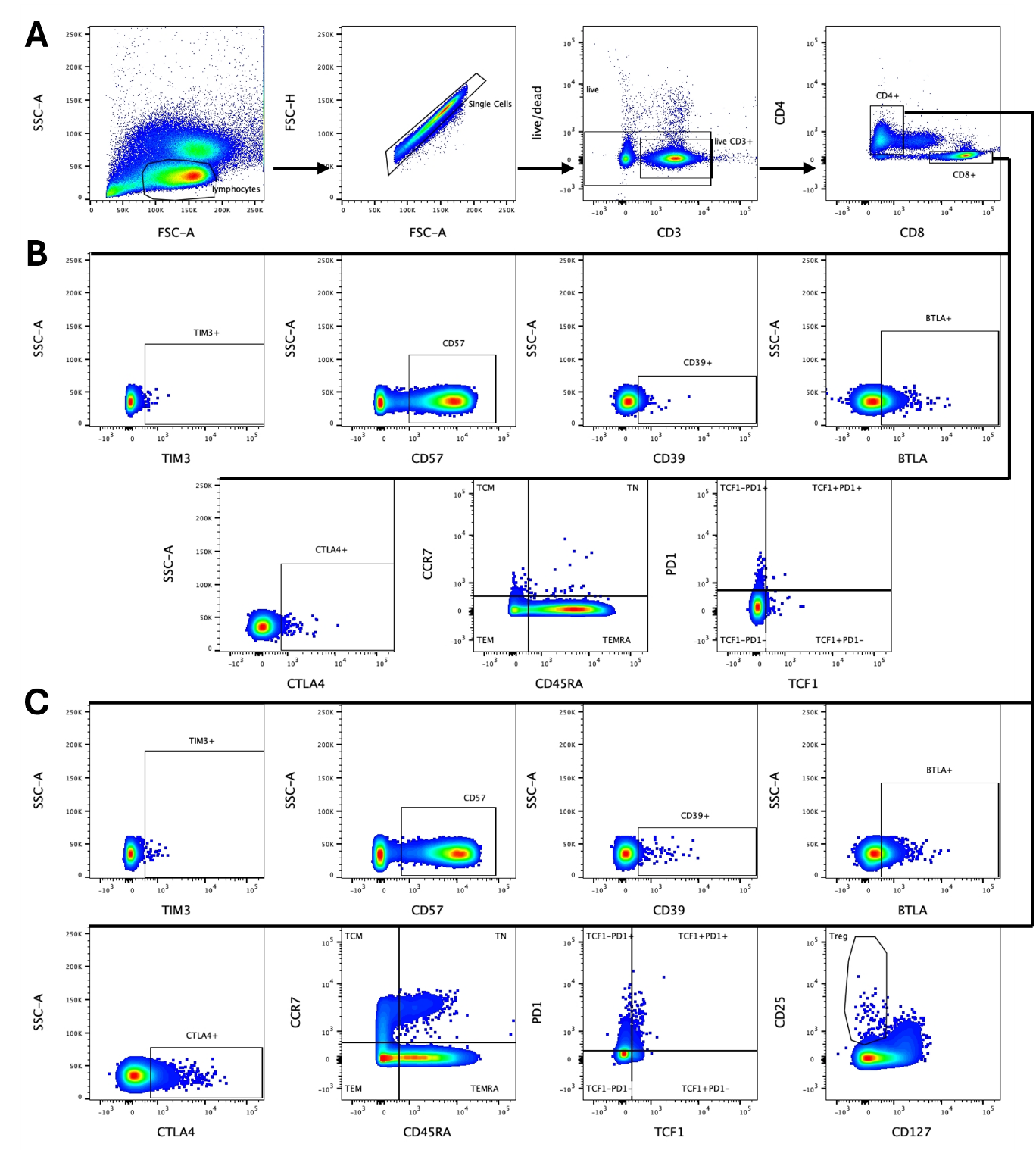
**

**Supplementary Figure 2: Exemplary gating strategy for markers of exhaustion on (B) CD8 and (C) CD4 T cell.** Gating of TCF1 and PD1 was performed based on FMOs. TIM3+ and TCF1-/PD1+ population was identified using Boolean gates. For FMO plots and compensation matrix refer to Supplemental Figure S1.


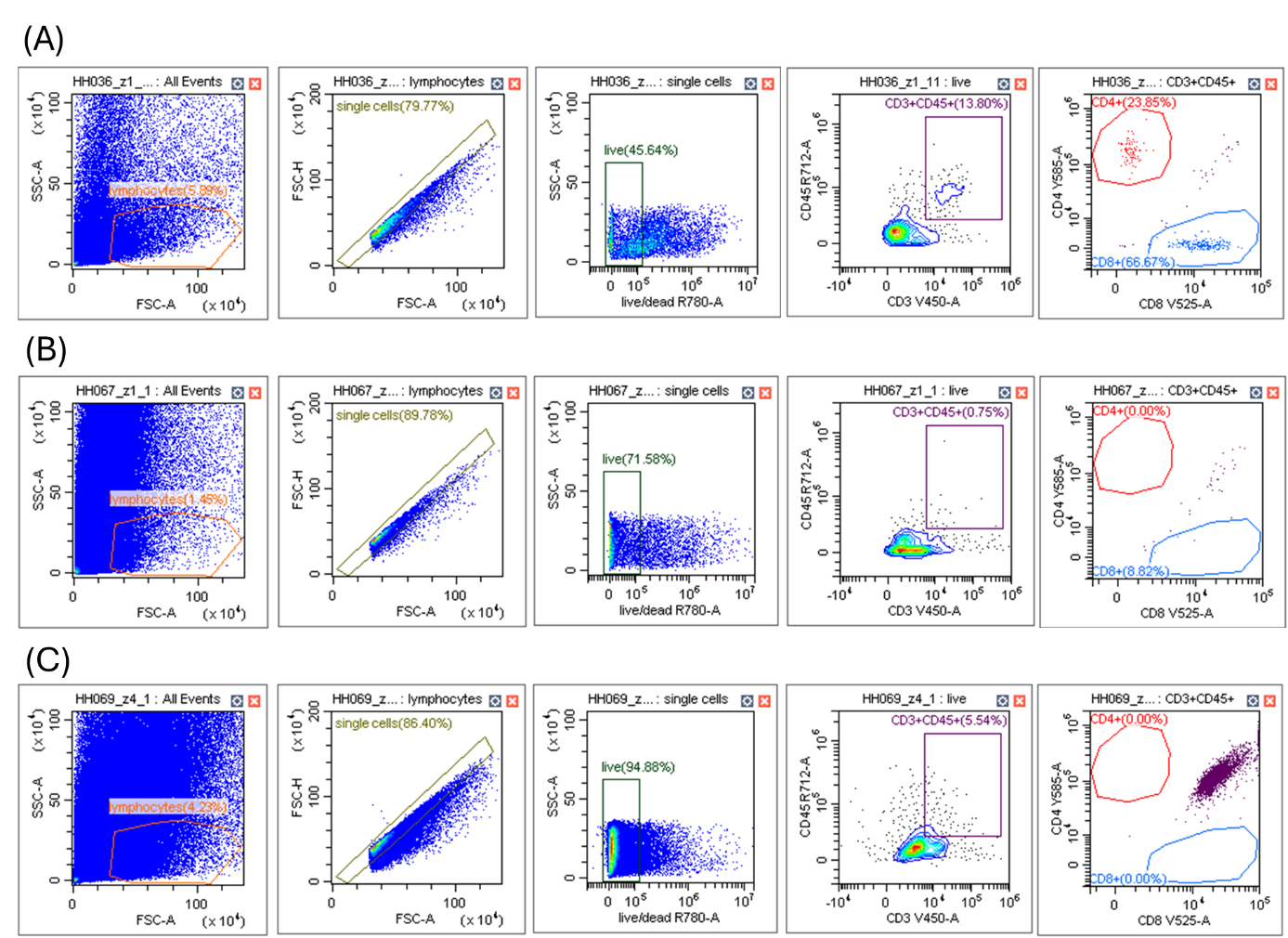


**
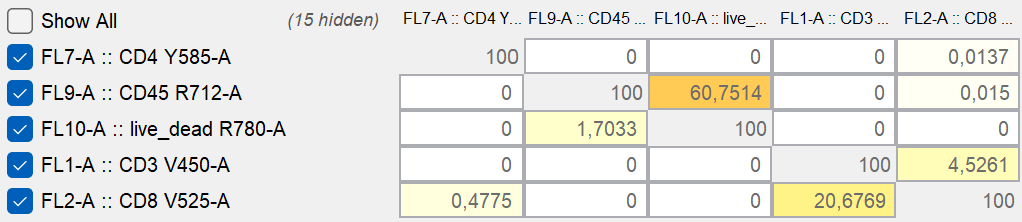
**

**Supplementary Figure 3:** Exemplary gating strategy for CD8 T cell sorting and compensation matrix.

**
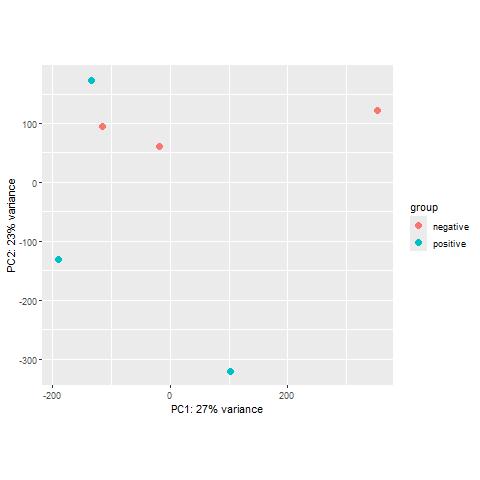
**

**Supplementary Figure 4: Principal Component Analysis (PCA) of individual bulk RNA-Seq samples.** PCA was performed on normalized gene expression data using 300 most variable genes. Each point represents one biological sample, colored according to experimental group. The axis show principal components 1 (PC1) and 2 (PC2) with respective total variance explained.

**
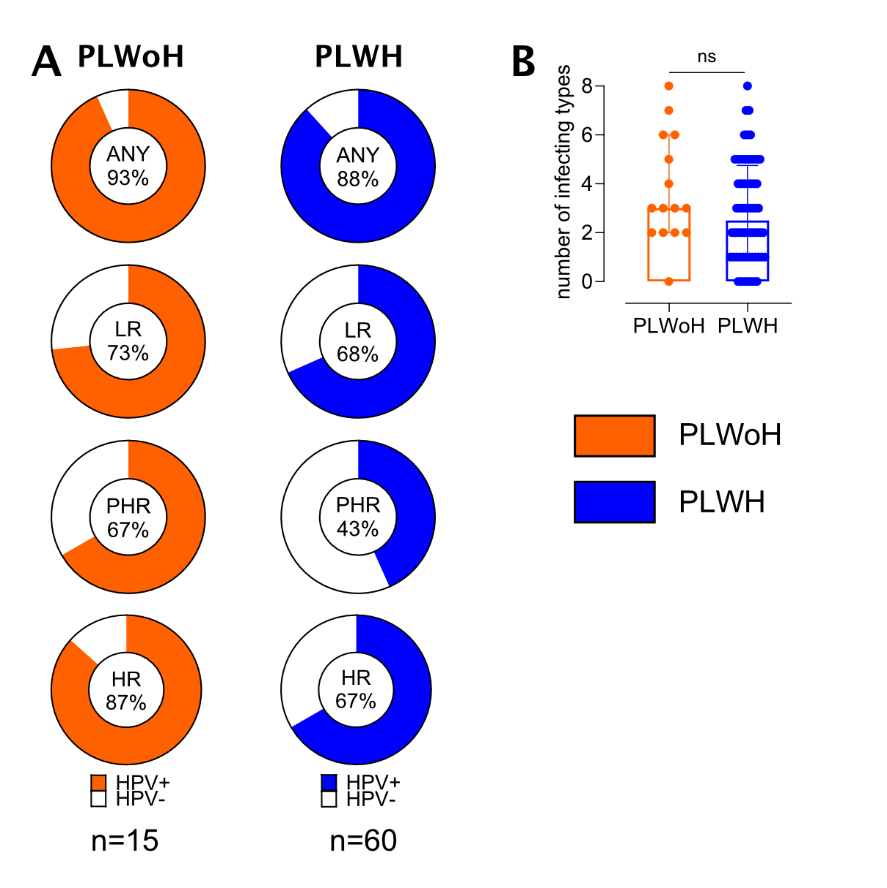
-**

**Supplementary Figure 5**: **HPV burden stratified by HIV status. (A)** Donut plots showing the percentage of individuals infected with any HPV type (ANY), low-risk (LR), probably and possibly cancerogenic types (PHR), or high-risk (HR) HPV types in people living without (orange, left, n=15) or with (blue, right, n=60) HIV at baseline. **(B)** Number of infecting HPV types per individual in men living without HIV (orange) compared to men living with HIV (blue) at baseline. Statistical significance was assessed using a Mann–Whitney test with an α level of 0.05.


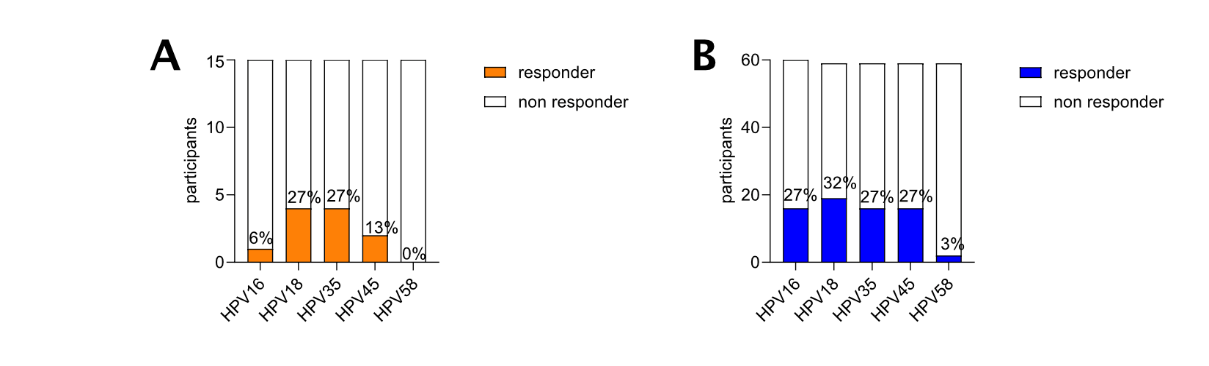


**Supplementary Figure 6: IFN-γ responses stratified by HIV status.** **(A)** Bar graphs show the percentage of men living without HIV (n = 15) exhibiting a positive IFN-γ response to each tested HR HPV genotype (16, 18, 35, 45, or 58) individually. **(B)** Same as (A) for men living with HIV (n = 60).

**
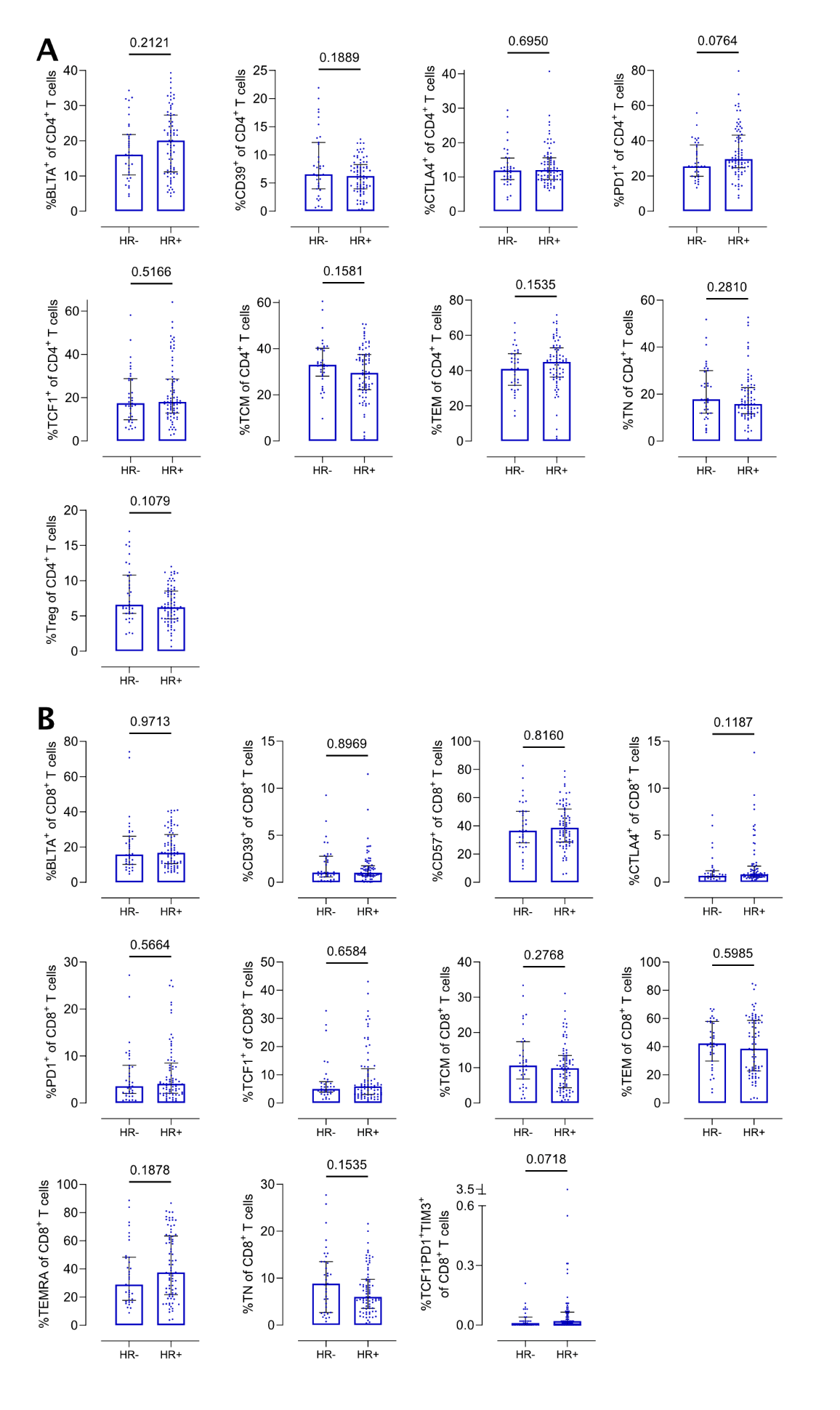
**

**Supplementary Figure 7: Markers of exhaustion and senescence. (A)** Frequency of CD4 T cells expressing exhaustion or senescence markers in men living with HIV with or without at least one HR HPV type. All graphs display the median with interquartile range. Statistical significance was determined using the Mann–Whitney test with an α level of 0.05. P values are indicated above the bar graphs. **(B)** Same as (A) for CD8⁺ T cells.
